# Searching for escape-resistant anti–SARS-CoV-2 neutralizing antibodies

**DOI:** 10.1172/JCI157416

**Published:** 2022-01-24

**Authors:** Ranjeet Singh Mahla, Lynn B. Dustin

**Affiliations:** Kennedy Institute of Rheumatology, University of Oxford, Oxford, United Kingdom.

## Abstract

A major goal of SARS-CoV-2 vaccination is the induction of neutralizing antibodies (nAbs) capable of blocking infection by preventing interaction of the SARS-CoV-2 Spike protein with ACE2 on target cells. Cocktails of monoclonal nAbs can reduce the risk of severe disease if administered early in infection. However, multiple variants of concern (VOCs) have arisen during the pandemic that may escape from nAbs. In this issue of the *JCI*, Jia Zou, Li Li, and colleagues used yeast display libraries to identify mAbs that bind to Spike proteins with a vast array of single amino acid substitutions. The authors identified mutation-resistant monoclonal nAbs for potential use as therapeutics. Multimerization further improved the potency of selected nAbs. These findings suggest a way forward in development of better nAb cocktails. However, the emergence of the highly mutated omicron (B.1.1.529) variant heightens the importance of finding effective anti–SARS-CoV-2 nAb therapeutics despite rapid viral evolution.

## SARS-CoV-2 and nAbs

**Figure 1:**
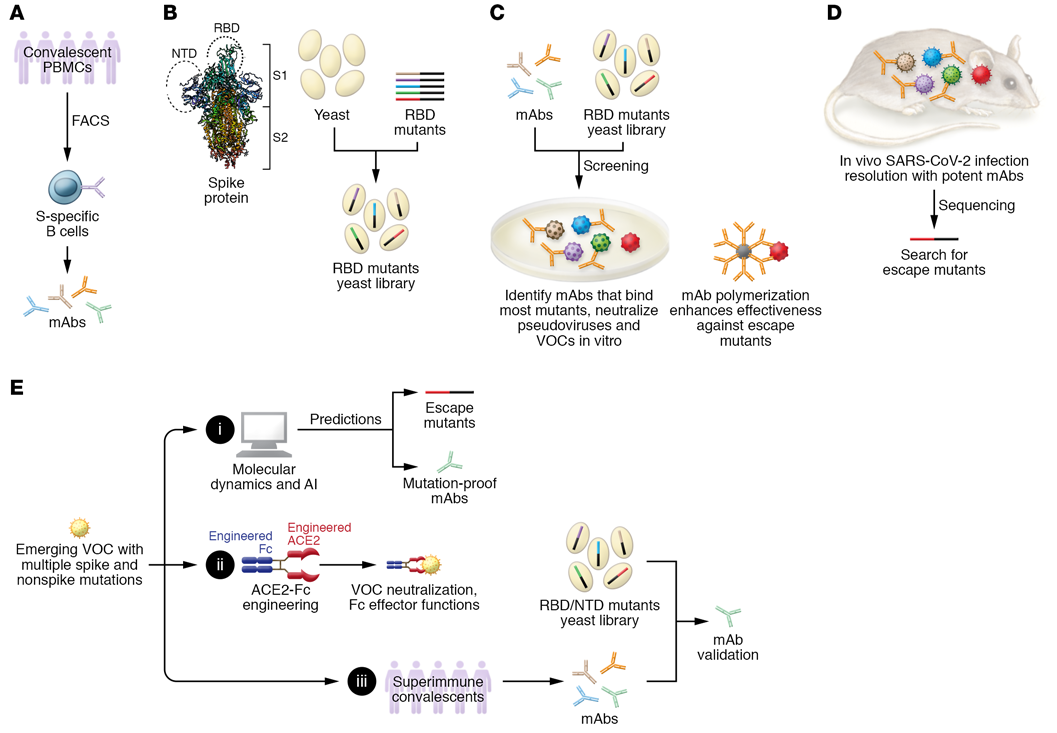
Development and validation of ultrapotent, mutation-resistant, neutralizing Abs as a multistep process. (**A**) Zou, Li, et al. (16) acquired and enriched Spike-specific (S-specific) B cells from subjects using FACS. Immunoglobulin heavy and light chains were cloned and expressed as mAbs. (**B**) Libraries of mutant S sequences were established for screening mAbs. (**C**) Potent mAbs bound with most mutants and neutralized pseudoviruses and variants of concern (VOCs) in vitro. mAb polymerization enhanced avidity to overcome some mutations that escaped neutralizing antibodies. (**D**) Similarly, potent mAbs reduced SARS-CoV-2 infection in vitro and in vivo. Escape mutants were identified by sequencing. (**E**) Ultrapotent mutation-resistant mAbs need to effectively neutralize emerging VOCs. Search strategies for screening and validation of escape-free mAbs could include the following: (i) Molecular dynamics to estimate binding free energy change, and AI to train and test mutation models, including replacement, insertion, and deletion mutations in the S receptor-binding domain (RBD) and N-terminal domain (NTD), and non-S proteins. These 2 strategies may identify potential escape mutants before they arise and serve as a reference for synthesis and validation of escape-free mAbs. (ii) Engineering of escape-free ACE2-Fc fusion proteins to directly neutralize VOCs while eliciting Fc effector functions. (iii) Sourcing mAbs from superimmune donors (those who had infection and later got vaccinated, or vice versa). Potential mAbs would require validation against complex RBD- and NTD-mutant libraries containing multiple mutations. The Spike protein structure shown was acquired from the Protein Data Bank (10.2210/pdb7LYO/pdb).

The SARS-CoV-2 Spike (S) glycoprotein is a major focus for vaccine and neutralizing antibody (nAb) development (Figure 1). S mediates viral binding to its cellular receptor, angiotensin-converting enzyme 2 (ACE2), and subsequent fusion of viral and endosomal membranes to deliver viral RNA into target cells. Homotrimers of S assemble on the surface of the viral particle, giving the virus its characteristic spiky or crowned appearance. Furin within the host cells cleaves S posttranslationally and the resulting fragments, called S1 and S2, remain noncovalently associated. The S1 domain includes the N-terminal domain (NTD) and the receptor-binding domain (RBD) responsible for binding to ACE2 on target cells. The S2 domain mediates viral fusion.

Potent SARS-CoV-2 nAbs have been cloned from rare S- and RBD-binding B cells in the blood of COVID-19 convalescent patients (1, 2). Monoclonal Abs (mAbs) with nAb activity have also been developed using humanized mice and through phage display technologies (1, 2). Most nAbs bind to epitopes on either the RBD or the NTD, and interfere with essential steps in virus entry. Many RBD-specific nAbs mask RBD’s ACE2-binding motifs (2) or sterically hinder RBD-ACE2 interactions. nAbs that bind to the NTD frequently target a single, positively charged supersite, and may block fusion at a postbinding step; their activity depends on Fc-mediated functions (3). Although mAbs could potentially target the fusion peptide or other critical structures on the S2 fragment, no potent S2-binding nAbs have yet been developed (2, 4). Several mAb cocktails have been developed for use in prophylaxis against, and passive immunotherapy of, COVID-19 (2, 4, 5). Prophylactic mAb cocktails may be particularly important in patients with compromised immunity. An additional goal is to identify nAbs active against other coronaviruses. Given that SARS-CoV-2 is the third highly pathogenic coronavirus to make the jump from animal reservoirs into humans in the past two decades, nAbs with broad anti-coronavirus function could be powerful weapons against a future pandemic.

## Keeping up with viral evolution

The rapid evolution of SARS-CoV-2 presents substantial challenges for both vaccination and development of therapeutic mAb cocktails. nAbs can mediate immunological selection for viral variants with reduced nAb binding. Importantly, immune responses targeting restricted viral epitopes enhance selection for viral escape through mutation of those same epitopes.

The omicron (B.1.1.529) variant of concern (VOC) has spread at breakneck speed since it was identified in late November, 2021. Omicron bears more than 50 amino acid substitutions, deletions, and insertions, including 30–37 coding changes in the S gene alone (exact numbers vary among the omicron sublineages already identified; ref. 6). These changes include many substitutions previously reported in the alpha (B.1.1.7), beta (B.1.351), and delta (B.1.617.2) VOCs as well as a host of changes not previously seen (6). Deletions within omicron’s NTD may knock out the epitopes for most NTD-specific nAbs (7). The omicron S protein has reduced sensitivity to polyclonal immune serum from COVID-19 convalescents (7–9) and recipients of SARS-CoV-2 vaccines (7–11). Mounting evidence shows that most mAb cocktails available to date, as well as those in advanced clinical development, have diminished ability to bind and neutralize the omicron variant (7–9, 11, 12).

With regard to VOCs, the search for universal anti-coronavirus mAbs remains active. Several research groups have sought mutation-proof mAbs to anticipate SARS-CoV-2’s next evolutionary move. Such mAbs would likely target conserved structures essential for S protein structure, receptor binding, and membrane fusion. Potent mAb cocktails should inhibit SARS-CoV-2 infection with effective concentrations in the picomolar range (ng/mL) or lower. Yeast display libraries have proven a powerful tool for evaluating RBD mutations and determining their effects on ACE2 and antibody binding (13–15).

In this issue of the *JCI*, Zou, Li, and colleagues identify human mAbs that can bind S bearing every possible single amino acid change at every possible site on the RBD (ref. 16 and Figure 1). The researchers started with convalescent PBMCs, and selected B cells with the ability to bind to both stabilized S trimers and S1. The somatically hypermutated immunoglobulin heavy and light chain gene variable domains were cloned and expressed as human IgG1 mAbs. mAbs with potent nAb activity were selected based on their ability to inhibit RBD-ACE2 binding, neutralize SARS-CoV-2 pseudoviruses, and neutralize authentic SARS-CoV-2 infection. mAbs were multimerized to enhance binding avidity, resulting in increased potency. RBD-mutant yeast display libraries were screened for the ability to bind to ACE2 but not to candidate mAbs, and the escape mutations were identified by sequencing. Commercial mAbs approved for use in the clinic (REGN10987, REGN10933, LY-CoV555, and LY-CoV016) were also tested against this RBD-mutant library, and served as benchmarks for comparison. This approach allowed the authors to identify low- and high-risk residues in the RBD. High-risk residues are potential mutation hotspots that tolerate substitutions with little loss of protein stability or ACE2 binding activity. Low-risk residues are conserved because changes are detrimental to protein stability or function. Consequently, SARS-CoV-2 nAb potency directly depends on counts of binding hotspot residues and corresponding mutational risk status. For example, as reported by Zou, Li, et al. (16), the nAb P15-16 was sensitive to substitutions at residue K378; however, K378-mutant pseudoviruses lost infectivity, making K378 a low-risk residue. nAb P5-22 was susceptible to mutations at F486, while nAb P14-44 was susceptible to mutations at K378, G381, P384, D427/428, and G413. Cocktails of P5-22 plus either P15-16 or P14-44 neutralized the SARS-CoV-2 VOCs alpha, beta, and delta, and were protective after establishment of infection in a mouse model. Crystal structures identified contact residues at which the nAbs bound to the RBD. Of note, however, omicron has 15 substitutions in its RBD relative to the ancestral Wuhan isolate of SARS-CoV-2 (6), plus numerous changes outside the RBD. These substitutions alter the size, polarity, and charge of contact residues as well as neighboring residues, with the potential for dramatic effects on nAb-epitope characteristics.

## Next steps

Like other viruses, SARS-CoV-2 evolves through error-prone replication and selective outgrowth of variants with improved fitness (17, 18). Changes in S may increase viral fitness by strengthening S-ACE2 interactions, leading to enhanced infectivity and transmissibility (18, 19), or — as potential hosts gain immunity through infection and vaccination — by destroying nAb epitopes (17, 18). Deleterious mutations in S may be rescued by compensating mutations at other sites within S (20) or other viral genes (epistasis; ref. 21). Perhaps truly mutation-proof nAbs would bind epitopes so structurally and functionally constrained that immune escape would be accompanied by total loss of infectivity. Methods are needed to predict the impact of immune selection on viral sequences, including the effects of complex, multiple amino acid changes. Several research groups are developing artificial intelligence (AI) approaches to anticipate the impacts of SARS-CoV-2 evolution (19, 22). Can AI predict groups of amino acid changes that may occur together, including compensating mutations (Figure 1)? Structural modeling predicts that mutations in omicron’s RBD lead to reduced binding by several well-studied nAbs (22). This prediction is supported by omicron’s apparent escape from immunity conferred by prior infection or immunization. Perhaps the most likely escape-proof nAb-like therapeutic would present a perfect mimic of the ACE2 surface, as seen by the RBD, or would provide a decoy for S to bind, such as an ACE2-Fc fusion protein (refs. 23, 24, and Figure 1). Superimmune individuals, who have encountered SARS-CoV-2 through both infection and immunization, may be recruited as donors to provide improved S-specific nAbs for cloning (Figure 1).

Zou, Li, and colleagues used a multipronged approach to screen nAbs for resistance to RBD mutations, develop an nAb cocktail targeting multiple RBD epitopes, and enhance nAb binding avidity for mutant RBDs (16). However, optimal therapeutic mAbs may need to go beyond neutralization of virus binding. The Fc domains of Abs link antigen recognition to the destructive powers of complement, phagocytic cells, and killer cells. Different immunoglobulin isotypes confer different effector functions. Antibody effector functions may directly relate to the efficacy of a therapeutic mAb cocktail (25). Multimeric structures, as presented by secreted IgM (or multimeric IgG; ref. 16), increase binding avidity despite weak interactions and might thus overcome reduced binding affinity. IgA provides protection at the respiratory surfaces where SARS-CoV-2 enters the body. A complementary approach might involve fusing ACE2-mimic decoys (23) with Fc domains specifying desired effector functions (ref. 24 and Figure 1). The next generation of nAb cocktails may need to target multiple epitopes (for example, RBD, NTD, and the fusion peptide) and direct multiple effector functions.

## Version 1 01/24/2022

In-Press Preview

## Version 2 02/15/2022

Electronic publication
